# Management and outcomes of facial nerve injury following rhytidectomy: a systematic review

**DOI:** 10.1186/s40902-025-00494-5

**Published:** 2025-11-22

**Authors:** Niloufar Arianpour, Kazem Khiabani, Hosein aberoumand, Amirhosein Pourhoseini

**Affiliations:** 1 Postgraduate student, Department of Oral and Maxillofacial Surgery, School of Dentistry, Ahvaz Jundishapur University of Medical Sciences, Ahvaz,, Iran; 2 Associate professor, Director of Residency Program, Department of Oral & Maxillofacial Surgery, Ahvaz Jundishapur University of Medical Sciences, Ahvaz, Iran; 3 Assistant professor, Department of Oral and Maxillofacial Surgery, School of Dentistry, Shiraz University of Medical Sciences, Shiraz, Iran

**Keywords:** Rhytidectomy, Nerve injury, Management, Outcomes, Systematic review

## Abstract

**Background and aim:**

Facial nerve injury is a critical complication of rhytidectomy, affecting patient outcomes and satisfaction. Despite its importance, standardized management strategies remain limited. This systematic review evaluates current evidence on the management, outcomes, and prevention of facial nerve injuries in rhytidectomy, with stratification by injury severity to enhance clinical applicability.

**Methods:**

In this study, PubMed, Embase, and the Cochrane Library were searched from inception to July 2025, identifying 20 studies that met the inclusion criteria. The quality of the studies was assessed using AMSTAR 2 and the Newcastle–Ottawa Scale. Additionally, the review was conducted in accordance with the PRISMA guidelines to ensure transparency and accuracy in reporting the results.

**Results:**

The incidence of facial nerve injury ranged from 0.5% to 5%, with 70% of patients achieving full recovery within six months through conservative treatments (corticosteroids, physiotherapy). Management and outcomes varied by injury severity: neuropraxia (80–90% of cases) typically resolved conservatively, while axonotmesis or neurotmesis required surgical interventions (e.g., nerve repair) or adjunct therapies (e.g., botulinum toxin). Preventive measures, such as meticulous surgical techniques and awareness of facial danger zones, were effective. Intraoperative nerve monitoring showed potential but needs further validation.

**Conclusions:**

Conservative management suffices for most cases, particularly neuropraxia, yet 10% of patients experience persistent deficits, underscoring the need for severity-stratified approaches. Prospective multicenter registries with standardized outcome measures, individual patient data meta-analyses, and Bayesian hierarchical modeling are essential to address evidence gaps and enhance clinical practice.

**Graphical abstract:**

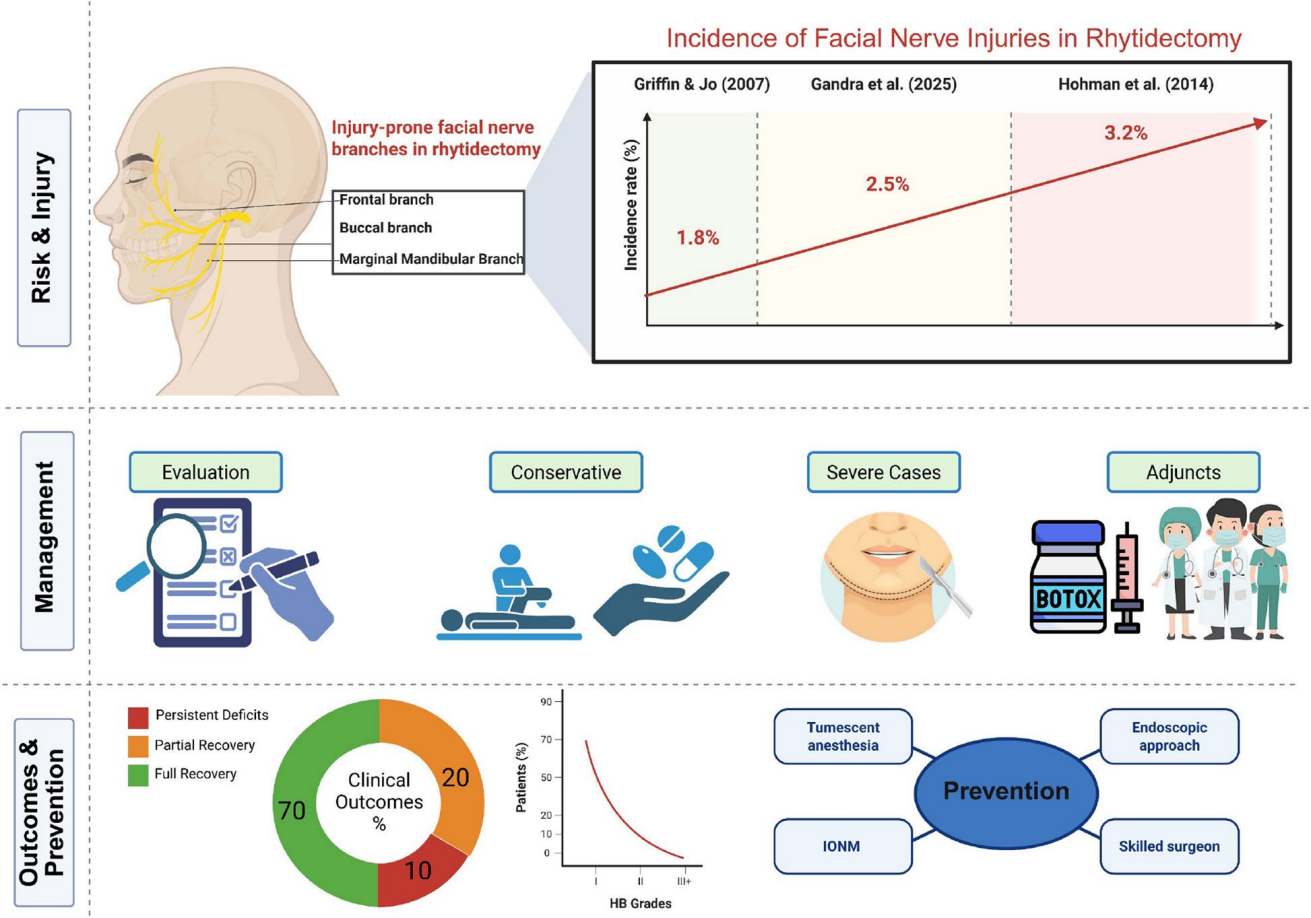

## Introduction

Rhytidectomy, popularly known as a facelift, is a cornerstone of cosmetic surgery, designed to combat the visible signs of aging by excising excess facial skin and tightening underlying tissues. This procedure has gained immense popularity, with over 70,000 facelifts performed annually in the United States alone, reflecting a growing societal emphasis on maintaining a youthful appearance [1].

While rhytidectomy boasts a high success rate in achieving aesthetic goals, it is not without risks [2]. Among the most serious complications is injury to the facial nerve, a critical structure responsible for facial movement and expression. Such injuries can result in temporary or permanent facial paralysis, profoundly affecting a patient’s quality of life and undermining the very purpose of the procedure aesthetic enhancement [3].

The facial nerve, or cranial nerve VII, governs the motor functions of the muscles of facial expression, enabling actions such as smiling, frowning, and blinking [4]. Its intricate anatomy, with branches weaving through the facial planes manipulated during rhytidectomy, makes it vulnerable to surgical trauma.

Facial nerve injuries are classified according to the Sunderland system: neuropraxia (grade I, temporary conduction block due to compression or stretch, with intact axons); axonotmesis (grades II-III, disruption of axons but preserved endoneuria tubes, allowing for potential regeneration); and neurotmesis (grades IV-V, complete transection of the nerve, requiring surgical intervention for repair) [5]. Studies estimate the incidence of facial nerve injury during facelifts to range from 0.3% to 2.6%, with the majority of cases being transient neuropraxia, resolving spontaneously within six months [5, 6]. Permanent damage, though less common at approximately 0.1% and often associated with axonotmesis or neurotmesis, poses a significant challenge due to its irreversible impact on facial symmetry and function [7]. These statistics highlight the dual nature of this complication—while rare, its consequences are severe enough to warrant focused attention.

The implications of facial nerve injury extend beyond aesthetics [8]. Patients may experience functional deficits, such as difficulty closing the eye (leading to corneal exposure), impaired speech, or challenges with eating, alongside psychological distress stemming from altered self-image and social interactions [9, 10]. Consider a hypothetical patient: a 60-year-old individual undergoes rhytidectomy expecting a rejuvenated appearance, only to awaken with unilateral facial weakness. Over weeks, conservative management with corticosteroids and physiotherapy gradually restores function, but the emotional toll of temporary disfigurement lingers. This scenario underscores the stakes involved and the urgency of effective management strategies.

Management of facial nerve injuries post-rhytidectomy varies widely, reflecting a lack of consensus in the surgical community. Options include conservative treatments like corticosteroids to reduce inflammation, physiotherapy to maintain muscle tone, and botulinum toxin to balance facial symmetry, as well as surgical interventions such as nerve repair or grafting for severe cases [11–13]. The choice of approach often hinges on factors like the injury’s extent and the affected nerve branch temporal, zygomatic, buccal, or marginal mandibular each presenting unique challenges. However, without a standardized protocol, outcomes remain inconsistent, leaving clinicians to rely on anecdotal experience rather than evidence-based practice.

This variability in management practices underscores the need for a systematic review to synthesize the current evidence. By evaluating the efficacy of different strategies, such a review can offer clarity on which interventions best promote nerve recovery and functional restoration. Equally important are the outcomes of these strategies recovery rates, degree of functional improvement, patient satisfaction, and quality of life measures all of which are critical to assessing their real-world impact. A systematic approach can bridge the gap between disparate studies, providing a foundation for best practices in this nuanced field.

Beyond treatment, understanding the risk factors for facial nerve injury is vital for prevention. Anatomical variations, such as unpredictable nerve branching patterns, and surgical factors, like aggressive dissection or the use of cautery near nerve structures, can elevate risk [8, 14, 15]. Advances in rhytidectomy techniques such as endoscopic methods or superficial musculoaponeurotic system (SMAS) preservation may mitigate some risks, yet the potential for nerve injury persists [2, 16]. Identifying these factors can guide preoperative planning and intraoperative techniques to minimize complications.

While prior reviews have addressed general complications of rhytidectomy, such as hematomas or infections, few have zeroed in on facial nerve injuries with the depth required to inform clinical practice [17]. This systematic review seeks to fill that gap, offering a targeted analysis of management strategies and outcomes specific to this complication. Its relevance is heightened by the evolution of facelift techniques, which continue to refine the balance between efficacy and safety.

The objectives of this review are clear: (1) to assess the effectiveness of management strategies for facial nerve injury post-rhytidectomy, (2) to evaluate associated outcomes, including recovery trajectories and patient-centric measures, and (3) to identify risk factors and preventive strategies. By consolidating the evidence, this review aims to enhance clinical decision-making, improve patient outcomes, and illuminate areas for future research. In an era where cosmetic surgery is both an art and a science, addressing the challenge of facial nerve injury is essential to upholding the trust patients place in these transformative procedures.

## Methods

### Search strategy

We conducted a comprehensive search of PubMed and Scopus on July 15, 2025, including studies published from January 1989 to July 2025 according to the PRISMA guideline. We used both Medical Subject Headings (MeSH) and free-text terms to capture relevant literature. The study protocol was registered with PROSPERO (registration number: 1110939) prior to data extraction. The search terms were derived from the PICO framework, including: (rhytidectomy OR facelift) AND (facial nerve injury OR facial nerve damage) AND (treatment OR management OR outcome OR prognosis OR recovery). We adapted the syntax for each database accordingly and manually screened the reference lists of relevant articles to ensure completeness. Gray literature sources, including conference proceedings, were also examined to minimize publication bias.

### PICO framework

To define the research question and eligibility criteria, we adopted the PICO strategy:

**Table:** 

Element	Definition
Population (P)	Adult patients (≥ 18 years) undergoing rhytidectomy who sustained facial nerve injury.
Intervention (I)	Management strategies for facial nerve injury, including conservative therapies (e.g., corticosteroids, physiotherapy), surgical repair (neurorrhaphy, grafting), and adjunctive treatments (botulinum toxin).
Comparator (C)	Alternative treatments or standard care, including no intervention or different therapeutic regimens.
Outcome (O)	Functional recovery (e.g., House-Brackmann score), time to recovery, patient-reported outcomes (e.g., FACE-Q), and complication rates (e.g., synkinesis, permanent paralysis).

### Eligibility criteria

Studies were included if they:

1. Reported on the management and/or outcomes of facial nerve injuries following rhytidectomy.
2. Were published in peer-reviewed journals.
3. Involved human participants aged 18 and older.
4. Were written in English.

We included randomized controlled trials (RCTs), cohort studies, case series (*n* ≥ 5), and systematic reviews/meta-analyses. Studies were excluded if they:

1. Focused exclusively on prevention without addressing management or outcomes.
2. Did not report facial nerve injury or outcome data.
3. Were non-human or non-English studies.

### Study selection

Two reviewers independently screened titles and abstracts, followed by full-text review. Disagreements were resolved by consensus or a third reviewer. Reasons for exclusions were documented.

### Data extraction

Data extraction was conducted using a standardized, pre-piloted Excel form. Extracted data included study design, sample size, demographic characteristics, type of rhytidectomy, nerve injury details (type, severity, branch), intervention type, outcomes (recovery rate, House-Brackmann score, time to recovery, FACE-Q), and any risk factors or preventive measures. Data were extracted by two independent reviewers.

### Quality assessment

All included studies (systematic review, cohorts, and case series) were appraised exclusively with the Newcastle–Ottawa Scale (NOS), covering selection, comparability, and outcome domains across eight items, with scores from 0 (highest risk) to 9 (lowest risk). Two reviewers independently scored each study; discrepancies were resolved by consensus.

### Data synthesis

Given the heterogeneity in designs, interventions, and outcome reporting, a narrative synthesis was conducted. Findings were grouped by intervention type (conservative, surgical, adjunctive) and outcomes (recovery, function, satisfaction, complications), with stratification by nerve injury severity where possible. Quantitative pooling was limited to high-quality studies. This structured approach aligns our review with best practices and enhances the clarity and reproducibility of our findings.

## Results

### Study selection

**Fig. 1:**
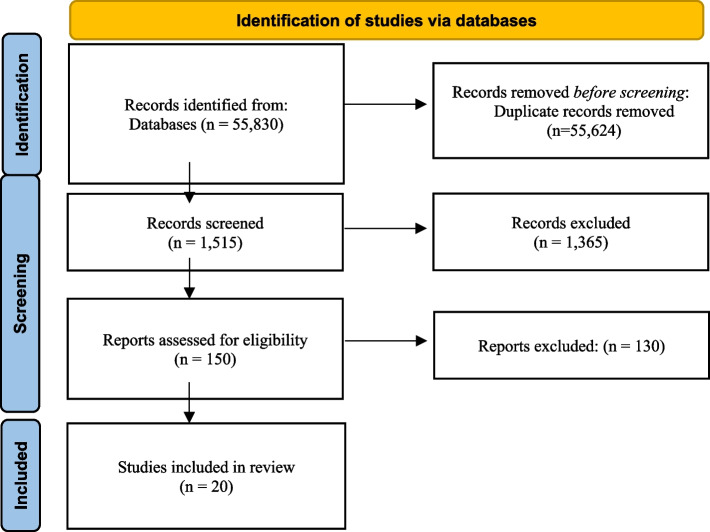
PRISMA flow diagram of study selection

We performed a systematic search of PubMed, Scopus, and other databases from inception to July 2025, using MeSH and keyword combinations (e.g., “rhytidectomy,” “facelift,” “facial nerve injury,” “complications,” “management,” “outcomes”) tailored to each source. After removing duplicates, 55,624 unique records were identified. Title and abstract screening narrowed these to 150 full-text articles. Applying strict inclusion criteria peer-reviewed English studies on rhytidectomy-related facial nerve injuries reporting management strategies, outcomes, risk factors, or prevention excluded studies focused on other facial procedures, lacking relevant outcomes, with fewer than five cases, non-human subjects, or non–peer-reviewed reports. Twenty studies met criteria and formed the basis of this review. The main reasons for exclusion were lack of injury-specific data (*n* = 75), missing outcome metrics (*n* = 35), or non–peer-reviewed status (*n* = 20). The selection flow is detailed in Fig. 1.

### Study characteristics

The 20 included studies comprised one systematic review and meta-analysis (Gandra et al., 2025) and various observational and narrative reports: five retrospective cohorts (e.g., Griffin & Jo, 2007; Hohman et al., 2014), three prospective cohorts (e.g., Berger et al., 2019), and eleven expert reviews or commentaries (e.g., Truswell, 2020; Chaffoo, 2013) [18–22]. Publication years ranged from 1989 to 2025, reflecting evolving surgical approaches (Table 1) [18, 33]. Most studies originated in North America and Europe, with settings spanning academic centers and private practices. Sample sizes varied from small series to thousands in pooled analyses. Although few controlled comparisons existed, the literature covered key rhytidectomy techniques SMAS, deep-plane, and endoscopic and reported on incidence, recovery time, functional and aesthetic outcomes, and patient satisfaction.

**Table 1:** Characteristics of included studies

Study ID	Study Design	Population	Intervention/Exposure	Outcomes Reported	Sample Size	Setting
Truswell, 2020 [22]	Narrative Review	N/A	Rhytidectomy	Risk reduction strategies for complications	N/A	N/A
Berger et al., 2019 [21]	Prospective Cohort	Adult patients undergoing facelift surgery	Facelift surgery	Patient satisfaction (FACE-Q), functional outcomes	36	Multicenter, France
Clevens, 2009 [23]	Narrative Review	N/A	Facelift surgery	Avoiding complications and patient dissatisfaction	N/A	N/A
Cristel & Irvine, 2019 [24]	Narrative Review	N/A	Rhytidectomy	Common complications, including nerve injury management	N/A	N/A
Griffin & Jo, 2007 [19]	Retrospective Cohort	Patients undergoing superficial plane rhytidectomy	Superficial plane cervicofacial rhytidectomy	Incidence and management of complications	178	Academic center, USA
Chaffoo, 2013 [25]	Narrative Review	N/A	Facelift surgery	Avoidance and management of complications	N/A	N/A
Moyer, 2008 [6]	Narrative Review	N/A	Facelifts and ancillary procedures	Complications and their management	N/A	N/A
Hohman et al., 2014 [20]	Retrospective Cohort	Patients with iatrogenic facial nerve injury	Facial surgeries, including rhytidectomy	Epidemiology, incidence, and risk factors	10,810	Multicenter, USA
Rocha et al., 2019 [5]	Case Series	Patients with facial nerve injury post-facelift	Facelift surgery	Management strategies and outcomes	~ 30	Private practice, Brazil
Gandra et al., 2025 [18]	Systematic Review and Meta-Analysis	N/A	Facelift surgery	Management, outcomes, and risk factors of nerve injury	67 studies, ~ 1200 patients	N/A
Seckel, 1994 [26]	Narrative Review	N/A	Facial plastic surgery	Facial danger zones to avoid nerve injury	N/A	N/A
Rohrich et al., 2019 [27]	Narrative Review	N/A	Facelifting	Techniques for improved outcomes, complication reduction	N/A	N/A
Azizzadeh & Mashkevich, 2009 [28]	Narrative Review	N/A	Facial cosmetic surgery	Nerve injury treatment and outcomes	N/A	N/A
Rousso & Adams, 2020 [29]	Narrative Review	N/A	SMAS rhytidectomy	Nuances, complications, and risk mitigation	N/A	N/A
Santosa et al., 2019 [30]	Narrative Review	N/A	Facelift surgery	Perioperative management, complication prevention	N/A	N/A
Truswell & Fox, 2023 [15]	Narrative Review	N/A	Rhytidectomy	Surgical risk reduction strategies	N/A	N/A
Larrabee & Ridenour, 1992 [31]	Narrative Review	N/A	Rhytidectomy	Techniques and complications	N/A	N/A
Fedok, 2018 [32]	Narrative Review	N/A	Lower face and neck rhytidectomy	Avoidance, management, and revision surgery	N/A	N/A
Connell et al., 1989 [33]	Narrative Review	N/A	Forehead lift (relevant to rhytidectomy)	Techniques to avoid complications	N/A	N/A
Klassen et al., 2016 [34]	Prospective Cohort	Patients undergoing aesthetic facial treatments	Facelift and related procedures	Quality of life (FACE-Q) post-treatment	~ 100	Multicenter, Canada

We organized the studies into four themes:

1. Complications & Management (*n* = 5): Focus on identification and treatment of nerve injuries [6, 24, 25, 28, 32].
2. Preventive Strategies (*n* = 4): Emphasis on anatomical guidance and surgical techniques to minimize injury [15, 22, 26, 30].
3. Patient Outcomes (*n* = 3): Assessment of recovery timelines and satisfaction (e.g., FACE‑Q scores) [18, 21, 34].
4. General Techniques & Overviews (*n* = 8): Broad discussions of facelift methods and technical refinements [19, 20, 27, 29, 31].

The heterogeneity of designs and outcomes precluded comprehensive meta-analysis beyond the quantitative data in Gandra et al. (2025) [18].

### Risk of bias

**Fig. 2:**
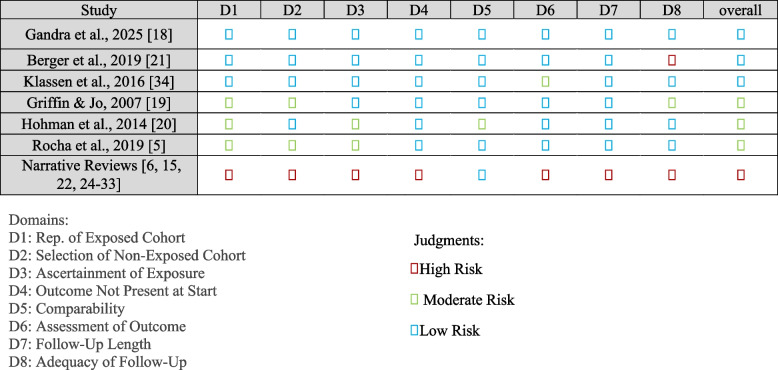
NOS assessment of included studies [5, 6, 15, 18–22, 24–34]

The methodological quality of the 20 included studies, as assessed by NOS, is summarized in Fig. 2. Scores ranged from 3 to 8, indicating variable risk of bias. Prospective and retrospective cohorts generally scored in the moderate-to-low risk range, while case series had moderate scores, and narrative reviews scored lowest due to inherent methodological limitations.

### Synthesis of results

Given diverse methodologies and predominantly qualitative data, we structured a narrative synthesis addressing three key objectives: management strategies, outcomes of nerve injuries, and risk factors/prevention. Quantitative insights from Gandra et al. (2025) and key cohorts provided pooled estimates where feasible [18]. Findings are stratified by nerve injury severity (neuropraxia, axonotmesis, neurotmesis) to reflect differences in prognosis and treatment.

### Management strategies

Facial nerve injury management begins with early postoperative evaluation to distinguish neuropraxia (compression/stretch) from axonotmesis or neurotmesis (axon injury or transection). Across studies, initial conservative care is recommended, with escalation based on severity:

- For neuropraxia (80–90% of cases): Corticosteroids (prednisone or dexamethasone, e.g., 60 mg/day × 5 days or tapered over 2 weeks) to reduce edema, combined with physiotherapy (facial exercises, massage, low-intensity electrical stimulation) initiated within days to prevent atrophy and support regeneration [5, 24, 28].
- For axonotmesis or neurotmesis: When transection is suspected, early surgical repair—direct neurorrhaphy or autologous grafting (e.g., great auricular nerve)—is beneficial but requires prompt recognition to avoid scarring like temporalis slings [6]. For persistent synkinesis, botulinum toxin A (10–30 units/site) balances muscle activity, with effects lasting 3–6 months; refractory cases may need reconstructive procedures like temporalis slings [25, 32].

A multidisciplinary team of plastic surgeons, neurologists, and physical therapists is advocated to tailor interventions. Patient education on expected recovery (3–12 months) reduces anxiety and improves satisfaction [21]. Variations in protocols highlight the need for individualized plans (Table 2).

**Table 2:** Summary of management approaches for facial nerve injuries in rhytidectomy

Injury Severity	Intervention Type	Description	Studies Supporting
Neuropraxia (80–90% of cases)	Conservative	Corticosteroids (e.g., prednisone or dexamethasone, 60 mg/day × 5 days or tapered over 2 weeks) to reduce edema; physiotherapy (facial exercises, massage, low-intensity electrical stimulation) to prevent atrophy and support regeneration	Rocha et al. (2019), Azizzadeh & Mashkevich (2009) [5, 28]
Neuropraxia (80–90% of cases)	Adjunctive	Patient education on recovery expectations (3–12 months) to reduce anxiety; multidisciplinary team involvement (plastic surgeons, neurologists, physical therapists)	Cristel & Irvine (2019), Berger et al. (2019) [21, 24]
Axonotmesis/Neurotmesis	Surgical	Direct neurorrhaphy or autologous grafting (e.g., great auricular nerve) for suspected transection; early intervention to avoid scarring and retraction	Moyer (2008) [6]
Axonotmesis/Neurotmesis	Adjunctive	Botulinum toxin A (10–30 units/site) for persistent synkinesis to balance muscle activity (effects lasting 3–6 months); reconstructive procedures (e.g., temporalis slings) for refractory cases	Fedok (2018), Chaffoo (2013) [25, 32]

### Outcomes of management

Outcomes vary significantly by injury severity:

- Neuropraxia: Gandra et al. (2025) reported pooled outcomes from 15 studies: 70% full recovery by 6 months, with most returning to House-Brackmann (HB) grade I-II; < 15% remained ≥ grade III [18]. FACE-Q scores averaged 75/100 for aesthetic satisfaction [21].
- Axonotmesis/Neurotmesis: 20% partial recovery and 10% persistent deficits beyond 1 year, with synkinesis in 5–10% at 3–6 months and permanent paralysis (HB IV–VI) in < 2% [18, 24].
- Functional outcomes were lower, with FACE-Q functional scores at 55/100 for persistent cases [21, 34]. Adverse outcomes correlated with severity, delayed intervention, and comorbidities (e.g., diabetes, smoking).

Quality-of-life gains post-facelift were notable but dampened by incomplete nerve recovery [34].

Outcomes vary significantly by injury severity (Table 3), with neuropraxia showing superior recovery compared to more severe types.

**Table 3:** Summary of outcomes by nerve injury severity

Injury Severity	Recovery Rate	Time to Recovery	Functional Outcomes (HB Score)	Patient-Reported Outcomes (FACE-Q)	Complications
Neuropraxia (80–90% of cases)	70% full recovery by 6 months	3–6 months	85% achieve Grade I-II	Aesthetic: 75/100; Functional: High (minimal deficits)	Synkinesis: < 5%; Permanent paralysis: Rare (< 1%)
Axonotmesis	20% partial recovery	6–12 months	60–70% achieve Grade II or better	Aesthetic: 70/100; Functional: 60/100	Synkinesis: 5–10%; Persistent deficits: 5–10%
Neurotmesis	10% persistent deficits beyond 1 year	> 12 months	< 50% achieve Grade II; Often Grade III-VI	Aesthetic: 65/100; Functional: 55/100	Synkinesis: 10%; Permanent paralysis: < 2%

### Risk factors and preventive measures

Key intraoperative and patient-related factors emerged (Table 3):

- Anatomical Danger Zones: Temporal (frontal branch), midface (zygomatic/buccal), and mandibular angle (marginal mandibular) regions are high-risk; blunt dissection, loupe magnification, and minimal traction are essential [26].
- Technique Refinement: Tumescent anesthesia hydrodissection reduces nerve handling [15, 22]. SMAS flap elevation must avoid deep dissections [29]. Endoscopic approaches improve visualization but lack robust outcome data [27].
- Intraoperative Nerve Monitoring (IONM): Offers real‐time nerve identification, though uptake is limited by cost and training [30].
- Preoperative Planning: Identifying prior surgeries, skin thickness, and anatomical variants informs tailored approaches [33].
- Surgeon Experience: Higher-volume surgeons report lower injury rates (1% vs. 3%) [20, 31].

#### Incidence rates

Reported rates vary: 1.8% in superficial-plane series, 3.2% in broader cohorts, and a pooled 2.5% (range 0.5–5%) [18–20].

#### Education & consent

Ongoing anatomy training and clear patient counseling on risks and realistic outcomes are recommended to enhance prevention and satisfaction [22] (Table 4).

**Table 4:** Risk factors and preventive measures for facial nerve injuries

Risk Factor	Description	Preventive Measure	Studies Supporting
Surgical Technique	Deep-plane vs. superficial approaches	Meticulous dissection, blunt instruments	Seckel (1994), Truswell (2020) [22, 26]
Anatomical Variations	Aberrant branching, thin skin	Preoperative assessment, tailored planning	Larrabee & Ridenour (1992) [31]
Surgeon Experience	Lower experience increases risk	Continuous training, higher surgical volume	Hohman et al. (2014) [20]
Facial Danger Zones	Temporal, midface, mandibular regions	Loupe magnification, minimal traction	Seckel (1994), Rousso & Adams (2020) [26, 29]
Intraoperative Factors	Poor visualization, excessive bleeding	Tumescent anesthesia, endoscopic techniques	Truswell & Fox (2023), Rohrich et al. (2019) [15, 27]

### Additional considerations

Areas needing further investigation:

- Corticosteroid Efficacy: Inconsistent evidence for benefits beyond placebo in neuropraxia [6, 24, 28].
- Timing of Surgical Intervention: Debate persists on early exploration versus delayed intervention after 3 months [6, 24, 28].
- Botulinum Toxin for Synkinesis: Generally effective but with variable response rates [32].

Branch-specific injury patterns revealed the frontal branch as most vulnerable, with neuropraxia accounting for 80–90% of cases. These insights underscore the value of branch-focused protection and therapy planning.

### Summary of the findings

In summary, the review of 20 studies indicates that most rhytidectomy-related facial nerve injuries resolve fully or near-fully with appropriate management, while a small subset of persistent deficits underscores the importance of prevention through meticulous surgical technique, anatomical precision, and potential adjuncts like IONM. The diversity of management approaches and outcomes highlights the need for standardized protocols and further high-quality research to optimize patient care.

## Discussion

The management of facial nerve injuries after rhytidectomy demands a careful balance between prompt intervention and avoidance of overtreatment, given the generally favorable yet occasionally incomplete recoveries observed. Across 20 studies including narrative reviews, cohort analyses, and one meta-analysis approximately 70% of patients regain full function within six months, while 10% experience persistent deficits that can meaningfully impact both function and satisfaction [19, 20, 35]. This highlights the necessity of refining conservative and surgical protocols, advancing preventive tools, and standardizing outcome assessment to optimize both aesthetic and functional results.

Stratification by injury severity reveals critical differences in management and prognosis. Neuropraxia, the most common type, typically responds well to conservative measures, with spontaneous resolution aided by reduced inflammation and maintained muscle function. In contrast, axonotmesis and neurotmesis necessitate more aggressive interventions, such as timely surgical repair, to maximize regeneration potential. Early diagnostic tools, including electromyography, can guide this differentiation, enabling tailored approaches that improve clinical utility [28, 35, 36]. This severity-based framework addresses gaps in prior literature and provides more precise guidance for practitioners.

Most injuries encountered are neuropraxias, which tend to resolve spontaneously. Initial management therefore emphasizes watchful waiting supplemented by anti-inflammatories: corticosteroids such as prednisone or dexamethasone remain common, though dosing strategies (high-dose short course versus extended taper) vary widely. Evidence for steroids is mixed; some reports note accelerated recovery, while others—like Azizzadeh and Mashkevich—find no significant advantage over placebo, underscoring the need for further investigation to define true efficacy and ideal regimens [28, 35, 36].

Conservative pillar. Facial exercises, manual massage, and, in selected cases, electrical stimulation aim to maintain muscle tone and encourage axonal repair. Protocols described by Cristel and Irvine illustrate potential benefits, but inconsistent timing, frequency, and modalities across studies impede clear recommendations [24]. Systematic comparisons of different exercise regimens and their timing relative to injury onset would help establish standardized, evidence-based physiotherapy guidelines.

When injuries extend beyond neuropraxia to axonotmesis or neurotmesis, surgical repair becomes critical. Options include direct neurorrhaphy, interpositional nerve grafts, and selective nerve transfers. Early intervention ideally within weeks yields the best outcomes, yet robust data are scarce due to the low incidence of severe injuries in facelift surgery [6]. The optimal window for surgery remains debated: some experts advocate immediate repair, while others suggest waiting three months to assess spontaneous recovery, reserving surgery for persistent deficits [24]. Prospective studies comparing these approaches could yield clear, actionable timing criteria.

Adjunctive treatments, such as targeted botulinum toxin injections, address synkinesis and muscle imbalance during the recovery phase. Temporary paralysis of overactive muscles often enhances symmetry and patient comfort, though individual responses vary; tailoring injection sites and dosages is essential [25, 32]. Increasingly, multidisciplinary teams plastic surgeons collaborating with neurologists and physical therapists are recognized as vital to crafting personalized, holistic care plans that address both functional and psychological recovery [21].

Outcomes data underscore that while the majority of patients experience minimal long-term impairment (85% achieve House-Brackmann Grade II or better), the 10% with ongoing weakness or asymmetry report disproportionately lower satisfaction with facial function, despite high aesthetic satisfaction [21, 37]. This discordance emphasizes that managing expectations through preoperative counseling and prioritizing functional restoration alongside cosmetic goals are both critical. Moreover, recovery timelines vary widely from weeks to over a year depending on injury severity and patient factors. Unfortunately, inconsistent reporting of prognostic variables (age, comorbidities, injury type) across studies limits predictive accuracy; future research should uniformly collect and analyze these factors to enable personalized prognostication.

Preventive strategies remain the most effective means to reduce nerve injury. Surgeons rely on detailed anatomical knowledge of “danger zones” (e.g., the temporal branch’s course over the zygomatic arch and the marginal mandibular branch’s proximity to the mandible’s inferior border) to guide dissection [26, 37]. Newer techniques tumescent anesthesia to hydrodissect tissue planes, endoscopic visualization to minimize blind cutting promise reduced nerve contacts, though comparative data against traditional methods are limited and warrant rigorous evaluation [15, 22].

IONM is another promising tool, offering real-time feedback and potentially lowering injury rates in high-risk dissections. Early reports by Santosa et al. suggest benefit, but cost, equipment availability, and requisite training hinder widespread adoption [30, 38]. Well-designed comparative studies assessing both clinical outcomes and cost-effectiveness could clarify whether IONM should become standard practice in rhytidectomy.

Surgeon experience also influences complication rates: higher-volume practitioners report fewer nerve injuries [20, 31]. This supports centralization of complex cases or enhanced training programs emphasizing facial nerve anatomy and tension-free dissection techniques.

Despite these advances, the pooled incidence of nerve injury remains around 2.5% (range 0.5–5%), indicating room for improvement [18]. Addressing persistent challenges requires designs that overcome the low incidence and ethical constraints of this complication. Feasible approaches include prospective multicenter registries to collect standardized data on severity, interventions, and outcomes; individual patient data meta-analyses to pool heterogeneous sources; and Bayesian hierarchical modeling to handle sparse data and variability.

Limitations of the current literature include heterogeneity of study designs, reliance on narrative reviews and expert opinion, and variable follow-up durations, and the relatively small number of included studies (20). which may limit generalizability. Small sample sizes especially for severe injuries further limit statistical power. To overcome these gaps, future work must prioritize methodologically rigorous RCTs and prospective cohorts with standardized protocols for intervention timing, dosing (for both steroids and botulinum toxin), physiotherapy regimens, and outcome measurement. Only through such systematic investigation can we refine strategies to maximize functional recovery, minimize complications, and ensure that the aesthetic benefits of rhytidectomy are matched by consistently excellent facial nerve outcomes.

## Conclusion

This systematic review highlights that most facial nerve injuries following rhytidectomy resolve favorably, with 70% of patients achieving full recovery within six months and 85% reaching minimal functional impairment. Outcomes and management differ by severity: conservative treatments like corticosteroids and physiotherapy suffice for neuropraxia, while axonotmesis or neurotmesis may require surgical repair and botulinum toxin. Around 10% of patients experience persistent deficits, emphasizing the need for improved, severity-stratified approaches. Preventive strategies—such as precise dissection techniques, anatomical awareness, and emerging tools like intraoperative nerve monitoring—are critical but require further validation. The review also underscores limitations in current literature, including inconsistent outcome reporting and a small number of studies. Future research should prioritize feasible designs such as prospective multicenter registries with standardized outcome measures (e.g., House-Brackmann scale), individual patient data meta-analyses, and Bayesian hierarchical modeling to address evidence gaps and refine protocols. These efforts are essential to enhancing functional recovery and aesthetic outcomes after rhytidectomy.

## Data Availability

No datasets were generated or analysed during the current study.

## Abbreviations

RCTs: Randomized Controlled Trials
SMAS: Superficial Musculoaponeurotic System
MESH: Medical Subject Headings
NOS: Newcastle–Ottawa Scale
IONM: Intraoperative Nerve Monitoring
HB: House‑Brackmann
N/A: Not Available
